# Improving assessment and escalation of threatened haemodialysis access: results of a nursing-led program

**DOI:** 10.1186/s12882-023-03321-z

**Published:** 2023-09-13

**Authors:** Dana Forcey, Dan Tran, Jenny Connor, Piriya Kusuma Na Ayudhya, Christian Ocampo, Craig Nelson, Sandra Crikis

**Affiliations:** 1 Department of Nephrology, Western Health, 176 Furlong Road, St Albans, VIC 3021 Australia; 2 Department of Medicine, Western Health, The University of Melbourne, Melbourne, VIC Australia; 3 Western Health Chronic Disease Alliance, St Albans, VIC Australia

**Keywords:** Haemodialysis, AV fistula, Fistula thrombosis, Nursing education program

## Abstract

**Background:**

Optimal vascular access is critical to successful haemodialysis. Acute thrombosis of haemodialysis access often leads to unplanned hospital admissions and interventions to restore patency.

Western Health is a large health service in Victoria, Australia. During the period February 2019 to January 2020, the rate of arteriovenous fistula (AVF) and arteriovenous graft (AVG) at Western Health satellite dialysis units was 0.33 episodes per 1000 patient-days, higher than the reported rate in the literature of 0.24 events per 1000 patient-days, and was associated with a cumulative total of 139 days of inpatient stay (2.2 per 1000 patient-days).

**Methods:**

The above results prompted creation of an education and escalation pathway for threatened haemodialysis access, based upon clinical markers of vascular access stenosis or imminent thrombosis assessed by nursing staff in satellite haemodialysis centres.

In the period February 2020 to January 2021, the education and escalation pathway was implemented. We assessed referrals via the pathway, rates of AVF/AVG thrombosis and associated hospital length of stay in the following 12-month period (February 2021 to January 2022).

**Results:**

Following introduction of the pathway, rates of AVF/AVG thrombosis declined to 0.15 per 1000 patient-days (*p* = *0.02)*, associated with a decline in attributable cumulative inpatient stay to 55 days (0.69 per 1000 patient-days).

**Conclusions:**

Our program demonstrates that the majority of thrombosed vascular access can be predicted and potentially averted with vigilant and well-practiced routine clinical assessment by trained nursing staff. Our nursing-led education and escalation program successfully identified vascular access at risk of imminent thrombosis, reduced rates of acute thrombosis and associated healthcare costs.

Despite these improvements, there are still disparities in outcomes for patients with thrombosed vascular access, with regards to length of stay and requirement for insertion of a temporary central venous catheter (CVC) for urgent dialysis whilst awaiting intervention, and these are areas for further investigation and improvement.

## Background

Optimal vascular access is critical for successful haemodialysis. The three types of permanent vascular access include arteriovenous fistula (AVF), arteriovenous graft (AVG) and permanent tunnelled central venous catheters. AVF is the preferred form of vascular access for patients undergoing long-term haemodialysis, [1] due to lower rates of healthcare-associated infection and mortality when compared to use of central venous catheters (CVC) [2, 3]. Arteriovenous grafts (AVG) are inferior to AVF for haemodialysis access in terms of infection risk and mortality; they remain superior to CVCs and are often placed when AVF are unsuitable for formation [4].

A major complication of AVF and AVG is acute thrombosis. Rates of thrombosed vascular access vary across health services and countries; a systematic review of fifteen studies from eight countries (USA, Canada, Australia, Taiwan, Italy, The Netherlands, Turkey, Iran) regarding AVF/AVG thrombosis (*n* = 4,232 fistulas) reported a median thrombosis rate of 0.24 events per 1000 patient-days [5]. Acute thrombosis of haemodialysis access often leads to unplanned hospital admission and interventions to restore patency, including mechanical or thrombolytic therapy via percutaneous catheter-directed thrombolysis, or surgical thrombectomy. Patients may require insertion of a CVC if they require urgent haemodialysis prior to restoration of AVF/AVG patency. The cumulative effect of delayed haemodialysis, invasive procedures, hospital admission and associated complications contributes to the increased risk of morbidity and mortality associated with thrombosed haemodialysis access [6].

Preoperative radiological vessel mapping, post-operative access surveillance, primary and secondary prevention pharmacotherapy and post-thrombosis thrombolysis, thrombectomy, angioplasty and stenting have all been investigated for the prevention of haemodialysis access thrombosis or recurrence [7]. Our study aimed to assess the impact of formalising nursing-led assessments of haemodialysis access and streamlining the escalation pathway for assessment of threatened haemodialysis access, and to quantify any associated impact on hospital admissions.

Western Health is a large tertiary health service that serves an ethnically, culturally and linguistically diverse population in Melbourne, Victoria, Australia. The health service has six haemodialysis units—four satellite and two in-hospital units. For the period from February 2019 to January 2020, the rate of thrombosed vascular access at Western Health satellite dialysis units was higher than the reported rate in the literature, at 0.33 episodes per 1000 patient-days. This finding, plus the increasing reliance on telehealth platforms rather than in-person clinical reviews during the COVID-19 pandemic highlighted a pressing need to upskill satellite dialysis nursing staff in their assessment of haemodialysis vascular access, streamline escalation pathways for threatened vascular access to ensure timely assessment and intervention.

To this end, an enhanced education and escalation pathway for threatened haemodialysis access was developed, based upon clinical markers of vascular access stenosis or imminent thrombosis [8]. The education program was implemented over a period of 12 months (February 2020 to January 2021), coupled with facilitating nursing referrals to a multi-disciplinary meeting of complicated vascular access. We assessed the rate of AVF/AVG thrombosis over the 12-month period following the program’s implementation, from February 2021 to January 2022.

## Methods

We retrospectively reviewed the number of acutely thrombosed AVF/AVG at Western Health and identified patients who underwent radiological or surgical intervention for acutely thrombosed haemodialysis access for the 12-month period from February 2020 to January 2021 by extraction of radiological procedural and discharge summary coding data. Demographic data and records regarding vascular access, treatment type and clinical outcomes were obtained by manual chart review and is displayed in Table 1.

**Table 1:** Prevalent haemodialysis patients’ demographics at Western Health as compared to prevalent haemodialysis patients in Australia [9]

	**Western Health** ***n* = 291** **n (%)**	**Australia* n* = 12,218** **n (%)**
**Age at first treatment**		
15–24	1 (0.3)	100 (0.8)
25–54	63 (21.6)	3006 (24.6)
55–74	138 (47.4)	5766 (47.2)
≥ 75	89 (30.6)	3346 (27.4)
**Gender**		
Male	186 (63.9)	7388 (60.5)
Female	105 (36.1)	4830 (39.5)
**Ethnicity**		
Aboriginal/ Torres Strait Islander	3 (1.0)	2014 (16.5)
Other	286 (98.3)	9943 (81.4)
Not reported	2 (0.7)	261 (2.1)
**Primary kidney disease**		
Diabetic kidney disease	152 (52.2)	4744 (38.8)
Glomerular disease	49 (16.8)	2334 (19.1)
Hypertension	42 (14.4))	1499 (19.1)
Polycystic kidney disease	12 (4.1)	663 (5.4)
Reflux nephropathy	1 (0.3)	329 (2.7)
Other	15 (5.2)	1797 (14.7)
Uncertain	20 (6.9)	673 (5.5)
Not reported		179 (1.5)
**Comorbid conditions**		
Current smoker	38 (13.1)	1530 (12.5)
Ex-smoker^a^	99 (34.0)	4344 (35.6)
Chronic lung disease	31 (10.7)	1754 (14.4)
Coronary artery disease	125 (43.0)	4504 (36.9)
Peripheral vascular disease	59 (20.3)	2300 (18.8)
Cerebrovascular disease	51 (17.5)	1431 (11.7)
Type 1 diabetes	14 (4.8)	516 (4.2)
Type 2 diabetes	184 (63.2)	6185 (50.6)
**Vascular Access**		
AVF^b^	236 (81.1)	9168 (75.0)
AVG^b^	6 (2.1)	464 (3.8)
Tunnelled catheter	48 (16.5)	2021 (16.5)
Non-tunnelled catheter	1 (0.3)	39 (0.3)
Not reported	0 (0)	526 (4.3)

^a^Patient self-identified, cessation of smoking for > 3 months

^b^*AVF* Arteriovenous fistula, *AVG* Arteriovenous graft

**Fig. 1:**
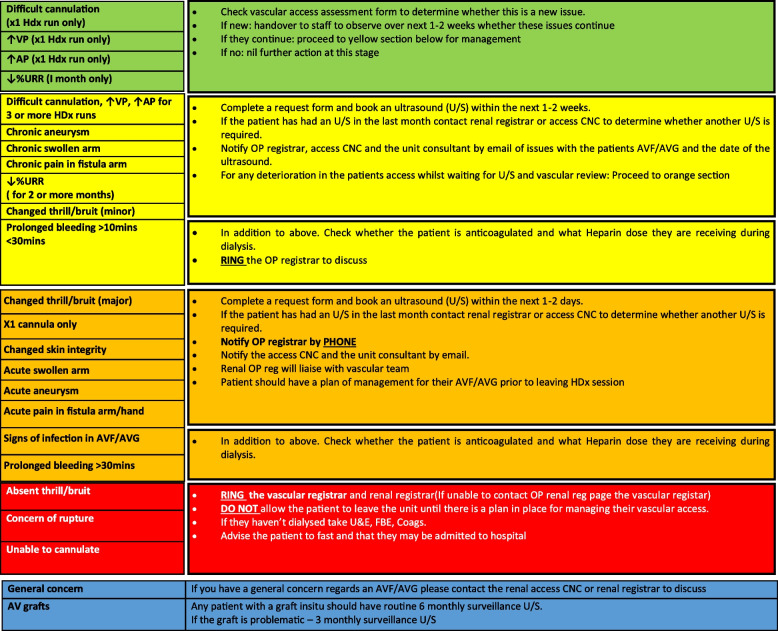
‘Traffic light’ escalation guideline and referral pathway HDx – Haemodialysis, AP – Arterial pressure, VP – Venous pressure, URR – Urea Reduction Ratio, CNC – Clinical Nurse Consultant, OP – Outpatient, U/S – Ultrasound, U&E – Urea and Electrolytes, FBE – Full Blood Examination, Coags – Coagulation studies, RING—Telephone

**Fig. 2:**
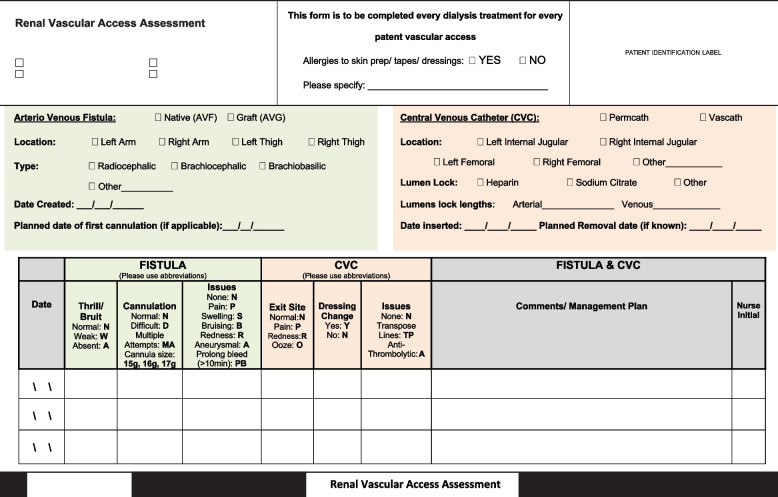
Vascular access assessment form for recording observations of patients’ haemodialysis access at each haemodialysis session

Following this review, an escalation pathway was developed for the recognition and escalation of threatened vascular access [8]. The pathway was colour-coded in a ‘traffic light’ system. (Fig. 1) Patients’ vascular access were assessed and observations recorded for each haemodialysis session (Fig. 2). In summary:

- Patients with one episode of difficult cannulation or elevated arterial/ venous pressures of > or < 180 mmHg, respectively, on a pump speed of < 300 ml/min, or patients with urea reduction rate (URR) reduced to < 65% were identified in the ‘Green’ pathway and were monitored for the following 1–2 weeks to determine if further action was required.
- Patients with difficult cannulation or elevated arterial/ venous pressures for ≥ 3 haemodialysis sessions, those with reduced URR for ≥ 2 months, those with chronic aneurysm, chronically swollen or painful AVF/AVG arm, minor change in bruit/thrill or prolonged bleeding (10–30 min) were identified via the ‘Yellow’ pathway, and arrangements made for doppler ultrasound assessment of vascular access within 1–2 weeks and notification to relevant medical and nursing staff.
- Patients who, on the side of their AVF/AVG had signs of infection, changed skin integrity, acute aneurysm, acute swelling or pain, or who had prolonged bleeding (> 30 min), major change in bruit/ thrill and those only able to cannulate for single-needle dialysis were identified via the ‘Orange’ pathway, and arrangements made for doppler ultrasound assessment of vascular access within 1–2 days with direct handover to medical and nursing staff.
- Patients with absent bruit/ thrill, unable to be cannulated for dialysis or concern for impending rupture of AVF/AVG were identified via the ‘Red’ pathway, with direct notification to medical staff. Patients were kept within the dialysis unit until plan arranged, and preliminary investigations were undertaken to guide urgency for haemodialysis.

An education program was delivered to nursing staff in the six haemodialysis units over the period February 2020 to January 2021. The education program consisted of a half-day education program from vascular surgeons, renal nurse educators and clinical nurse consultants regarding AVF/AVG formation, interpretation of ultrasound and fistulagrams, and an in-depth introduction to the traffic light education and escalation pathway. This was followed up by a consolidation education session regarding the traffic light education and escalation pathway two months later to all haemodialysis satellites, delivered over an online platform.

Haemodialysis nursing staff were also able to refer patients to a multi-disciplinary meeting to review complicated vascular access. This meeting was attended by senior interventional radiologists, Vascular Surgeon, Nephrologists, vascular access nurses and nursing educators, haemodialysis observations and radiological images were reviewed and any recommended actions for complicated vascular access was advised.

We collected data regarding thrombosed AVF/ AVG over a 12-month period following the implementation of the education and escalation pathway (February 2021 to January 2022). In addition to the clinical data collected from the pre-intervention period, we also collected:

- Time from presentation to hospital to intervention to thrombosed vascular access
- Use of systemic anticoagulation whilst awaiting intervention

Thrombosis rates and cumulative days of inpatient stay were calculated as incidence rate ratio per 1000 patient-days and compared using χ^2^. Data were analysed using Stata version 13 (Stata statistical Software: Release 13.0. College Station TX: Stata Corporation), and statistical significance was set at *p* < *0.05*.

We also reviewed all angiographic fistula examinations and percutaneous balloon angioplasties of AVF/AVG referred during the post-intervention period, to identify those referred via the escalation pathway (‘orange’ pathways).

We excluded home haemodialysis patients and those patients who had thrombosis of AVF/AVG prior to haemodialysis commencement via this vascular access, as this study aimed to assess the effect of the education program delivered to satellite haemodialysis nurses specifically assessing in-use vascular access.

This project was granted institutional ethics from the Western Health ethics department (QA2022.73, ERM-90919).

## Results

### Patient population

The patient characteristics and vascular access of prevalent haemodialysis patients at Western Health as compared to the Australian population [9] is shown in Table 1*.*

### Pre-intervention period

During the period February 2019 – January 2020, there were 217 patients dialysing at a satellite haemodialysis centre—168 via AVF, 8 via AVG. There were 26 acute thromboses of AVF/AVG in 18 individual patients (0.33 episodes per 1000 patient-days). 14 episodes of acute thrombosis occurred in native AVFs and four in AVGs. The median age of the thrombosed vascular access was 3 years (Inter-quartile range (IQR) 6 months – 10 years). 17 episodes of acute thrombosis (65%) occurred in vascular access that had previously thrombosed and undergone intervention and one (4%) in vascular access that had previously undergone intervention for stenosis without thrombosis. 8 episodes (31%) occurred in vascular access that were not known to have any prior issues and had not previously required intervention.

31% (*n* = 8) of episodes of acute thrombosis were preceded by clinical signs of stenosis of imminent thrombosis but were not escalated and actioned; two episodes (8%) had relevant clinical signs that were escalated, imaged and were awaiting intervention. 61% (*n* = 16) of episodes of acute thrombosis of vascular access did not have any preceding clinical signs of stenosis of imminent thrombosis.

15 interventions were performed by interventional radiology and 11 were performed surgically. In 80% of cases (*n* = 21), intervention successfully restored patency of the AVF/AVG. 15 patients (55%) required the insertion of a temporary CVC (including both non-tunneled and tunneled dialysis catheters) whilst awaiting intervention to their thrombosed vascular access. The median length of stay after an episode of thrombosed vascular access was four days (IQR 2- 6 days), and there was a cumulative total of 139 days in hospital for patients due to acutely thrombosed vascular access (2.2 per 1000 patient-days). There were no related patient deaths.

### Post intervention follow-up period

During the post intervention period (February 2021 – January 2022), there 251 patients dialysing in satellite haemodialysis centres – 192 via AVF, 8 via AVG. There were 14 episodes of acute vascular thrombosis (0.15 per 1000 patient-days). Each episode of vascular thrombosis occurred in individual patients; none experienced recurrent thrombosis of their vascular access during this period. 13 thromboses occurred in AVFs and one in an AVG. The median age of vascular access was 2.4 years (IQR 11 months—5yrs). Four episodes of acute thrombosis (29%) occurred in vascular access that had previously thrombosed and undergone intervention, five (36%) in vascular access that had previously undergone intervention for stenosis without thrombosis and one in an AVF with a known > 50% stenosis in vessel lumen on ultrasound that had not previously compromised dialysis and for which the patient had declined intervention. Four (29%) were not known to have any prior issues and had not previously required intervention. With regards to sustained hypotension as a risk factor for thrombosis, one patient had intra-dialytic hypotension [10] during the haemodialysis session preceding thrombosis and one patient had a surgical procedure during which an episode of hypotension was recorded in the week prior to thrombosis. The remaining 12 patients did not have episodes of sustained hypotension documented in their preceding haemodialysis sessions.

Thirty-nine patients were referred for fistulagrams and/or fistulaplasties via ‘yellow’ or ‘orange’ pathways, including: recirculation (*n* = 8), high venous pressures (*n* = 5) and prolonged bleeding after dialysis (*n* = 2). 35 (90%) of these patients did not experience any acute thrombosis and were successfully managed as day cases via interventional radiology. Four patients (10%) experienced thrombosis of the known at-risk vascular access that had already been escalated and imaged with doppler ultrasound and were awaiting angiographic intervention. Their time delay from ultrasound to thrombosis ranged from 3 days to 1 month (median 2 weeks).

Of the remaining ten patients with thrombosed vascular access who had not been referred by the escalation pathway, two had increased venous pressures on dialysis prior to thrombosis, which met criteria for escalation via the ‘orange’ pathway but were not escalated accordingly. Eight patients had no indications for referral via the escalation pathway. Table 2 shows the clinical details of vascular access that thrombosed during this study period.

**Table 2:** Clinical details of thrombosed vascular access before and after program implementation

	**Pre intervention** **n (%)**	**Post-intervention** **n (%)**	***p-value***
**Access type**			
AVF^a^	14 (78%)	13 (93%)	0.24
*Radio-cephalic*	*6 (43%)*	*9 (69%)*	*-*
*Brachio-cephalic*	*8 (57%)*	*4 (21%)*	*-*
AVG^a^	4 (22%)	1 (7%)	0.45
**Known issue with vascular access/ prior intervention of same**			
No	8 (31%)	4 (29%)	0.88
Yes	18 (69%)	10 (71%)	-
- *Nil intervention*	*0 (0%)*	*1 (6%)*	-
- *Treatment of stenosis*	*1 (4%)*	*5 (36%)*	-
- *Treatment of thrombosis*	*17 (65%)*	*4 (29%)*	-
**Signs of threatened haemodialysis access prior to thrombosis**			
No	16 (61%)	8 (57%)	0.79
Yes	10 (39%)	6 (43%)	-
- *Not escalated*	*8 (31%)*	*2 (14%)*	-
- *Escalated and imaged, awaiting intervention*	*2 (8%)*	*4 (29%)*	-

^a^*AVF* Arteriovenous fistula, *AVG* Arteriovenous graft

Following presentation to hospital with thrombosed haemodialysis access, an attempt to salvage the thrombosed vascular access was made in all cases, all initially undertaken percutaneously by interventional radiology, after a median time from admission to intervention of 24 h (range 2 h—6 days). Longest delays to intervention were experienced by those patients admitted on Fridays and on weekends. Percutaneous radiological intervention successfully restored patency to the vascular access in 11 cases (79%). One percutaneous radiological intervention was unsuccessful secondary to patient refusal to continue the procedure rather than technique or procedural failure. After failed percutaneous intervention, two patients proceeded to vascular surgical intervention after a further 24 h—8 days; surgery did not successfully restore patency to the vascular access in either case.

Patients who required insertion of a temporary CVC for urgent dialysis (*n* = 4) waited a median of 4 days to radiologic intervention, compared to 2 days waited by those who did not require insertion of temporary access. Whilst awaiting intervention, nine patients were treated with systemic anticoagulation (heparin infusion in all instances); five patients did not any receive systemic anticoagulation.

**Fig. 3:**
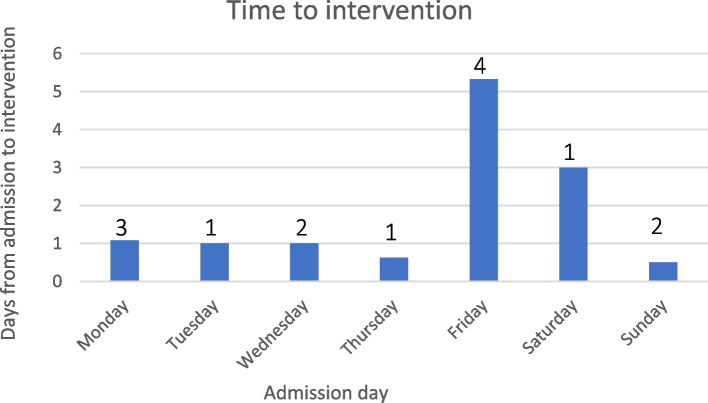
Mean time (days) from admission to interventional radiological intervention, by admission day. Number of patients admitted on each day of the week listed above graph bars

The median length of hospitalisation attributable to thrombosed vascular access was three days (IQR 2—5.5 days), and there was a cumulative total of 55 days of attributable inpatient stay due to acutely thrombosed vascular access (0.69 per 1000 patient-days). There were no related patient deaths. Table 3 shows the outcomes for patients admitted with acutely thrombosed vascular access in the post-intervention study group (*n* = 14) and Fig. 3 shows the time to radiological intervention for these patients, by admission day.

**Table 3:** Outcomes for patients admitted with acutely thrombosed vascular access after implementation of the escalation and education program

	**n (%)**
**Anticoagulation**	
*No*	5 (46%)
*Yes – Heparin infusion*	9 (64%)
*Yes – Other*	0 (0%)
**Temporary dialysis catheter insertion**	
*No*	10 (71%)
*Yes*	4 (29%)
**Restoration of access patency**	
*No*	3 (21%)
*Yes*	11 (78%)

## Discussion

This study demonstrates that a nursing-led education program and escalation pathway can facilitate early identification of threatened haemodialysis access and successfully reduce rates of acute thrombosis. Key strengths of this study are its multi-disciplinary care model and the analysis of hospitalisation rates and inpatient lengths of stay changed after implementation of the program, from which cost savings can be estimated.

Haemodialysis nurses identify the acquisition of technical skills of haemodialysis and responsibility for prolonging patients’ lives as central to their work, [11] and nurses’ knowledge and guideline-based management of haemodialysis vascular access improves practice patterns and patient outcomes [12]. Our program worked closely with nursing staff to identify the barriers to escalation of threatened vascular access. This informed the development of the escalation algorithm to streamline the referral process, both for imaging and intervention, and for discussion in a multi-disciplinary radiology meeting of complicated vascular access. The education program was delivered with the unit’s dedicated renal vascular access nurse, a role which has been demonstrated to improve patient outcomes [13].

After the education and escalation program was implemented, there was a reduced rate of acute thrombosis of haemodialysis access from 0.33 to 0.15 episodes per 1000 patient-days (*p* = *0.02),* with an associated reduction in attributable inpatient admission rate from 2.2 to 0.69 per 1000 patient-days.

The education and escalation program referred an additional 35 patients for angiographic assessment and intervention for ‘orange’ indications during the post-intervention period, which may have averted thrombosis of these vascular access. Although there could be additional costs conferred by undertaking intervention on these vascular accesses with ‘orange’ indications which may not have ultimately thrombosed, our escalation program utilised observations during haemodialysis paired with clinical assessment of vascular access as the basis for referral via the escalation pathway and not upon the basis of findings of stenosis alone, in keeping with current guidelines discouraging routine imaging for AVF/AVG stenosis [1]. Our program was demonstrated to be an effective and inexpensive way to reduce rates of thrombosis of haemodialysis access, without significant increase in investigations yielding non-significant findings.

A range of triggers for escalation were incorporated into and prompted escalation via the pathway, indicating that the parameters included in the pathway were appropriate. The most common triggers for escalation via the yellow/orange pathways included recirculation, high venous pressures and prolonged bleeding. All patients referred via the pathway who ultimately developed acute thrombosis of their vascular access were referred for elevated venous pressures and this remains a key clinical parameter indicating threatened vascular access.

Two patients with thrombosed haemodialysis access after the implementation of the escalation pathway had increased venous pressures on dialysis prior to thrombosis meeting criteria for escalation via the ‘orange’ pathway but were not escalated accordingly. This highlights that the traffic light system can successfully identify haemodialysis access at risk of imminent thrombosis, but ongoing education and regular reminders are required to ensure staff utilise the pathway effectively to avert acute thrombosis of haemodialysis access.

Two patients with thrombosed AVF after the implementation of the escalation pathway had documented hypotension prior to thrombosis, one episode of intra-dialytic hypotension [10] and one during a surgical procedure. Sustained hypotension is a risk factor for AVF thrombosis, [14] and further measures to enhance nursing and patient monitoring of vascular access after hypotensive episodes may be beneficial.

In order to identify opportunities for improvement beyond the implementation of the escalation pathway, data were collected from the post-intervention period regarding time from imaging to thrombosis and thrombosis to intervention, as well as utilisation of systemic anticoagulation whilst awaiting intervention. Four patients were escalated via the pathway, confirmed to have threatened vascular access and were awaiting angiographic assessment at the time of thrombosis. Though these patients all underwent doppler ultrasound assessment within the recommended timeframe 1–2 days after referral via the ‘orange’ pathway, a median time period of 2 weeks (range 3 days—one month) elapsed from ultrasound-confirmation of threatened haemodialysis access to thrombosis. Whilst the traffic light escalation pathway has defined recommended time frames from escalation to imaging, further work is required across multiple departments to establish appropriate guidelines regarding the optimum time or acceptable time delay from ultrasound-confirmation of threatened haemodialysis access to intervention, in order to avert acute thrombosis.

Following presentation to hospital with thrombosed haemodialysis access, the majority of patients underwent timely angiographic intervention; longest delays to intervention were experienced by those patients admitted on Fridays and on weekends. Patients who required insertion of a temporary CVC for urgent dialysis waited a median of 4 days to radiologic intervention, compared to 2 days waited by those who did not require insertion of temporary access. There is reduced staffing across the hospital service after hours and on weekends, with interventionalists working in an on-call capacity. Further work is required to streamline access to timely intervention to reduce rates of insertion of temporary vascular access, and possibly improve rates of salvage of thrombosed haemodialysis access.

Finally, there was noted variability in the administration of anticoagulation for thrombosed haemodialysis access. Subsequent guideline modification has made routine application anticoagulation for the inpatient management of thrombosed haemodialysis access now standard in our health service.

## Conclusion

Acute thrombosis of haemodialysis access places patients at significant risk of morbidity associated with hospitalisation, procedural intervention and delayed haemodialysis. These events also confer a significant cost burden to the healthcare system by way of unplanned admissions, emergency surgical or radiological procedures and insertion of temporary vascular access. This study highlights that a nursing-led education and escalation program can successfully identify vascular access at risk of imminent thrombosis, reduce rates of acute thrombosis and reduce associated healthcare costs. A significant proportion of haemodialysis vascular access thrombosis can be predicted and potentially averted with vigilant and well-practiced routine clinical assessment by trained nursing staff and streamlined escalation pathways. More work is needed to ensure that patients with threatened haemodialysis access receive assessment and intervention in a timely manner to avert acute thrombosis, and to restore patency to vascular access when thrombosis occurs, in order to provide best patient care.

## Data Availability

The data that support this case report are available from Western Health, but restrictions apply to the availability of these data, due to patient confidentiality. Data are however available from author Jenny Connor upon reasonable request and with permission of the Western Health Ethics Board.

## Abbreviations

AVF: Arteriovenous fistula
AVG: Arteriovenous graft
CVC: Central venous catheter
IQR: Inter-quartile range
URR: Urea Reduction Ratio
AP: Arterial pressure
VP: Venous pressure
CNC: Clinical Nurse Consultant
OP: Outpatient
